# “The value of pre- and co-seasonal sublingual immunotherapy in pollen-induced allergic rhinoconjunctivitis”

**DOI:** 10.1186/s13601-015-0061-z

**Published:** 2015-05-04

**Authors:** Pascal Demoly, Moises A Calderon, Thomas B Casale, Hans-Jørgen Malling, Ulrich Wahn

**Affiliations:** Allergy Division, Pulmonology Department, Hôpital Arnaud de Villeneuve, University Hospital of Montpellier, Montpellier, France; Sorbonne Universités, UPMC Paris 06, UMR-S 1136 INSERM, IPLESP, Equipe EPAR, Paris, France; Section of Allergy and Clinical Immunology, Imperial College London-NHLI, Royal Brompton Hospital, London, UK; Internal Medicine, Morsani College of Medicine University of South Florida, Tampa, FL, Omaha, NE USA; Allergy Clinic, Gentofte University Hospital, Copenhagen, Denmark; Department of Paediatric Pneumology and Immunology, Charité Virchow-Klinikum, Humboldt University, Berlin, Germany

**Keywords:** Allergic rhinitis, Pre-seasonal, Co-seasonal, Sublingual immunotherapy, Pollen, Birch, Grass

## Abstract

Allergen immunotherapy (AIT) is a guidelines-approved, disease-modifying treatment option for respiratory allergies, including allergic rhinitis (AR) induced by pollen. The various AIT regimens employed to date in pollen-induced AR can be classified as continuous (i.e. year-round) or discontinuous (i.e. pre-seasonal alone, co-seasonal alone or pre- and co-seasonal). Pre-and co-seasonal regimens are typically used for sublingual allergen immunotherapy (SLIT) and have economic and compliance advantages over perennial (year-round) regimens. However, these advantages must not come at the expensive of poor efficacy or safety. The results of recent double-blind, placebo-controlled, randomized clinical trials show that pre- and co-seasonal SLIT is safe and effective in patients with AR induced by grass pollen (treated with a tablet formulation) or by birch pollen (treated with a liquid formulation). Progress in SLIT has been made in defining the optimal dose of major allergen, the administration frequency (daily), the duration of pre-seasonal treatment (four months) and the number of treatment seasons (at least three). Post-marketing, “real-life” trials of pre- and co-seasonal birch or grass pollen SLIT regimens have confirmed the efficacy and safety observed in the clinical trials. In the treatment of pollen-induced AR, pre- and co-seasonal SLIT regimens appear to be at least as effective and safe as perennial SLIT regimens, and are associated with lower costs and good compliance. Good compliance may mean that pre- and co-seasonal SLIT regimens are inherently more effective and safer than perennial SLIT regimens. When considering the pre- and co-seasonal discontinuous regimen in particular, a 300 IR five-grass-pollen formulation is the only SLIT tablet with a clinical development programme having provided evidence of short-term, sustained and post-treatment efficacy.

Allergic rhinitis (AR) is a chronic, aeroallergen-induced, immunoglobulin E (IgE)-mediated inflammatory disease of the upper airways that affects up to 30% of adults and up to 40% of children, and has an impact on asthma [1-3]. Allergic conjunctivitis is a comorbidity in around two-thirds of people with pollen-induced AR. It is now accepted that AR has a considerable disease burden, with a marked socioeconomic impact and negative effects on health-related quality of life, work and school performance, sleep, mood and social functioning [4-10]. The pollen released by wind-pollinated plants (including grasses, trees and weeds) for one or more periods of several weeks or several months each year is a major atopic sensitizer in the general population in Europe and North America [11,12]. Seasonal AR is associated with well-defined pollen periods, the start date and duration of which vary from year to year and as a function of the geographic and climatic zone [13,14]. For example, the duration of Poaceae pollen seasons recorded at 13 European pollen-monitoring stations varied from 69 days in Reykjavik to 154 days in Thessaloniki [13]. Birch (*Betula*), olive (*Olea europaea*) and cypress (*Cupressus* sp.) trees also produce highly allergic pollens [14]. The duration of the Betulaceae pollen season varies from 38 days in Reykjavik to 107 days in Legnano (near Milan) [13]. Importantly, exposure to pollen (and thus induction of clinical disease in atopic subjects) is increasing as a result of climate change, with higher pollen counts, longer pollen periods and less rainfall in many regions [14-16].

## Allergy immunotherapy (AIT) for pollen-induced AR

Most cases of AR are treated with symptomatic, anti-allergic medications (such as H1-antihistamines) and/or anti-inflammatory medications (such as intranasal corticosteroids (ICSs)) [1,2,17,18]. However, around 30% of patients (most of whom have moderate-severe AR) do not gain sufficient disease control with symptomatic medications [19]. AIT is a guidelines-approved, disease-modifying treatment option for IgE-mediated respiratory allergies [1,2,20-23]. The effect of AIT on symptoms is at least as great as that produced by ICSs [24,25]. As a disease-modifying treatment, AIT is clinically effective for up to 12 years [26,27] and reduces the appearance of new sensitizations and allergic asthma in patients with AR [28]. Both subcutaneous immunotherapy (SCIT) and sublingual immunotherapy (SLIT) are effective in AR, according to the results of large-scale, double-blind, placebo-controlled (DBPC) randomized clinical trials (RCTs), position papers and meta-analyses [27,29-35]. Furthermore, SLIT preparations can be administered as drop or tablet formulations [20,21,36-39]. Despite the proven short-term and long-term efficacy of both SCIT and SLIT and decades of clinical experience, there is still little consensus on (i) the optimal dose of desensitizing allergen (due to the low number of dose-ranging studies for SLIT solutions), (ii) the administration frequency, (iii) the overall treatment duration, (iv) the treatment regimen, and (v) the seasonality of AIT in pollen-induced AR [31,34].

## Administration regimens for the treatment of pollen-induced AR

**Figure 1:**
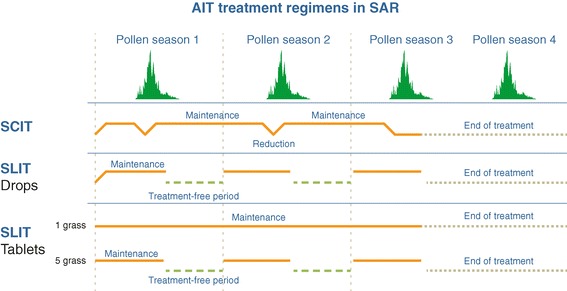
**AIT regimens for seasonal allergic rhinitis.**

In AIT, multiyear administration regimens (Figure 1) can be dichotomized as continuous (i.e. year-round) or discontinuous (i.e. with a treatment-free period each year).

Setting aside the allergen build-up phase (during which administration of the maintenance dose of allergen is achieved over a number of weeks, days or even hours, depending on whether conventional, rush or ultra-rush protocols are applied), SCIT is generally administered via a continuous, year-round maintenance regimen in which injections performed in a medically supervised setting at regular intervals (typically every 4 to 6 weeks for aeroallergen extracts) for several years and thus over several pollen periods (Figure 1) [22]). Injections with longer time intervals have not been tested in DBPC RCTs. The literature data suggest that the risk of a severe adverse reaction to pollen SCIT is exacerbated during the corresponding peak pollen period [40]. For example, Lockey et al.’s analysis of data collected between 1945 and 1973 found that 41% of the recorded SCIT-related deaths had occurred during the pollen period [41]. Hence, the latest US practice parameter for AIT confirms historical practice and suggests that maintenance dose levels may be reduced (and certainly not increased) during periods when the patient is naturally exposed to high levels of the disease-inducing allergen [22,42,43] (Figure 1).

Despite the proven efficacy of SCIT, a number of safety and ease-of-use concerns remain. Indeed, SLIT has advantages in terms of safety and convenience (especially in children) [20,21,44]. Although pre-seasonal-only, co-seasonal-only and perennial regimens have all been evaluated for SLIT formulations, a pre- and co-seasonal regimen is most commonly used (Figure 1). A 2009 review of pollen SLIT administration regimens (mostly involving drop formulations) [45] listed three studies with a pre-seasonal-only regimen (e.g. [46]), three with a co-seasonal-only regimen (e.g. [47,48]), eight with a continuous or partially post-seasonal regimen (e.g. [49]) and 27 with a pre- and co-seasonal regimen. Historically, pollen SLIT drops have been administered daily, every other day or three times a week. In pollen-induced AR, continuous or post-seasonal administration is challenging because patients will not perceive the benefit of SLIT after the pollen period has ended (i.e. when their symptoms have disappeared). Longer periods of medication are associated with poorer compliance and thus a lower likelihood of effectiveness [50,51].

Conversely, pre- and co-seasonal regimens also have limitations. Trials of a five-grass pollen SLIT tablet formulation have shown that 2 months of pre-seasonal treatment (followed by co-seasonal treatment) is less effective than 4 months of pre-seasonal treatment [52,53]. Similarly, a trial of a single-grass pollen SLIT tablet formulation found that the magnitude of the reductions in rhinoconjunctivitis symptom and medication scores increased with the duration of preseasonal treatment (4 months appeared to be optimal) [54]. Hence, a patient with a pollen allergy following a pre- and co-seasonal regimen from one year to another must remember to obtain medication and initiate treatment long enough ahead of the pollen season. This particular problem (persistence of treatment) is avoided if a year-round regimen is strictly adhered to. In a study in Germany, the prescription renewal rate (a proxy for treatment persistence) for SLIT or subcutaneous immunotherapy formulations were higher (at 55%–71%) than those reported elsewhere for conventional medications [55].

A large body of evidence attests to the safety and efficacy of pre- and co-seasonal regimens for pollen SLIT drops when administered for one season or several consecutive seasons. Recently, Worm et al. performed a robust, two-season, DBPC RCT of pre- and co-seasonal treatment with a 300 index of reactivity (IR) birch pollen sublingual solution in 574 adult immunotherapy-naïve patients with birch-pollen-induced allergic rhinoconjunctivitis at 56 investigating centres in 11 European countries [56]. The treatment started 4 months before the expected pollen period. At the start of the study, 68% of the patients were polysensitized, 20% had mild-to-moderate asthma and 54% had oral allergy syndrome. The primary efficacy endpoint over the second birch pollen period) was found to be significantly lower (p < 0.0001) in the active SLIT group (relative difference vs. placebo: 30.6%; difference in the least-squares (LS) means: −2.04 [95% confidence interval: −2.69; −1.40]). A 19.0% difference was also seen for active SLIT in the first pollen period. The average medication score was significantly lower in the 300 IR SLIT group in both seasons, with relative LS mean differences of −29.3% and −41.9%, respectively (p < 0.0001 for both). Active treatment was associated with a better Rhinoconjunctivitis Quality of Life Questionnaire (RQLQ) score in the first and second pollen periods, with relative LS mean differences of −23.1% and −34.5%, respectively. Treatment-emergent adverse events (TEAEs) were no more frequent in the 300 IR SLIT group than in the placebo group and were less frequent in the second season (respectively 70.7% and 64.0% of patients affected in season 1, and 46.8% and 48.6% p in season 2. This decrease over time was also observed for common, local adverse events (AEs) such as oral pruritus (24.7% of patients affected in season 1 and 8.3% affected in season 2 in the SLIT group; 3.8% and 0.8% in the placebo group, respectively. The treatment’s efficacy and the frequency of TEAEs did not appear to depend on the presence or absence of oral allergy syndrome [56].

In a multicentre, DBPC, randomized phase III trial, Wahn et al. [57] randomized 207 children aged between 4 and 12 with grass pollen AR/rhinoconjunctivitis with or without bronchial asthma into an active treatment group (SLIT with an aqueous six-grass pollen extract) and a placebo group. A pre- and co-seasonal regimen was applied. The primary efficacy end point was the comparison of the change of the area under the curve for the symptom–medication score, starting from the baseline season to the first grass pollen season after the initiation of treatment. The values were -212.5 and -97.8 in the active SLIT group and the placebo groups, respectively (p < 0.004), evidencing a significantly greater reduction in severity in the active SLIT group. In terms of safety, 75.9% of the patients in the active SLIT treatment group experienced at least one AE (compared with 32.7% of the patients in the placebo group) but no treatment-related severe AEs were recorded. However, only one pollen period was studied; the effect of treatment discontinuation and resumption could not therefore be assessed.

Although most studies of pre- and co-seasonal SLIT regimens have dealt with grass and birch pollen formulations, other pollens have been studied. For example, pre- and co-seasonal SLIT with a standardized aqueous ragweed pollen extract was studied by Creticos et al. [58] A total of 429 patients were randomized (active SLIT: n = 218; placebo: n = 211) into a multicentre, DBPC, randomized, phase III trial in North America. The treatments were administered between April 2011 (i.e. at least 4 months before the start of the ragweed pollen period in October) and November 2011 (the end of the period). The primary efficacy end point was the average daily rhinoconjunctivitis symptom-medication score over the course of the ragweed pollen season; the value in the SLIT group (0.82 ± 1.64) was significantly lower than in the 1.44 ± 2.40 in the placebo group (p < 0.001). Again, only one pollen period was studied and the effect of treatment discontinuation and resumption was not assessed.

Although DBPC RCTs remain the gold standard for evaluating investigational products, “real-life” studies are essential for completing effectiveness and safety under “real-life” conditions in the target patient population. To this end, Hadler et al. performed an open, prospective, non-interventional study in Germany evaluating the 300 IR birch pollen SLIT solution in 716 polyallergic and monoallergic patients (mean ± SD age: 38 ± 16; age range 3-87) over two pollen seasons [59]. A pre- and co-seasonal regimen was applied. The patients’ symptoms were scored on a hybrid scale that took account of disease severity and frequency for rhinitis alone, conjunctivitis alone and rhinoconjunctivitis. The mean rhinitis score in the birch season prior to the study was 4.83. This value fell to 3.17 in the first pollen season and 2.31 (a 52% reduction, p < 0.001) in the second pollen season. The mean conjunctivitis score fell from 3.74 to 2.1 and then 1.69 (a 55% reduction, p < 0.001). Lastly, the mean rhinoconjunctivitis score fell from 3.76 to 2.29 and then 1.76 (a 53% reduction, p < 0.001). In a subgroup analysis, the 300 IR pollen SLIT solution appeared to be similarly effective in mono-allergic and polyallergic patients and in those with an intra-seasonal start to treatment. The percentage of patients requiring symptomatic medication was also significantly lower (p < 0.001) during the first and second pollen season (59% and 48%, respectively, compared with 81% before the study. Both mono-allergic and poly-allergic patients gained clinical relief. The 300 IR birch pollen SLIT solution was well tolerated, and 97% of the patients evaluated tolerability as “good” or “very good”. Ninety percent of the patients did not report an adverse event during the two treatment seasons [59]. In clinical practice, the majority of patients receiving sublingual immunotherapy with allergen-specific 300 IR SLIT solution are satisfied with treatment [60], and compliance and prescription renewal rates are relatively high [55,61].

Taken as a whole, these “real-life” findings for 300 IR birch pollen SLIT solution confirmed the results of the DBPC RCTs.

The third main type of AIT formulation is the SLIT tablet. Two tablet formulations of grass pollen SLIT (a single-grass tablet [36,37] and a five-grass pollen tablet [38,39]) have been approved to date for an indication of grass-pollen-induced AR (with or without conjunctivitis) in adults and children. According to the respective summaries of product characteristics and the pivotal DBPC RCTs, a 75,000 standardized quality tablet (SQ-T) single-grass SLIT tablet [36,37] is approved for administration with a continuous, year-round regimen (starting before the first pollen season; Figure 1), whereas a 300 IR five-grass SLIT tablet [38,39] is approved for administration with a discontinuous pre-and co-seasonal regimen (i.e. starting four months before the expected start of the pollen season and finishing at the end of the pollen season; Figure 1). Hence, the total treatment duration per year is between 5 and 7 months (depending on the duration of the pollen season) and the onset of action (in an allergen challenge chamber study) was found to occur after one month of treatment [62].

Over the last 10 years, the safety and/or efficacy of pre-seasonal and co-seasonal treatment with a novel, 300 IR five-grass-pollen SLIT tablet has been unambiguously demonstrated in a series of pre- and post-marketing clinical trials in adult and paediatric populations [39]. Worldwide, a total of 2,512 study participants have been randomized to receive either the five-grass-pollen SLIT tablet (n = 1,514) or a placebo (n = 998) [39]. Furthermore, the 300 IR five-grass-pollen SLIT tablet was proven to be similarly effective in several clinical profiles (monosensitized patients, polysensitized patients, patients with high symptom scores and patients with high skin sensitivity) [63]. After the pivotal, single-season Phase II/III DBPC RCT in which the dose of 300 IR was selected for further investigation and registration [64], the 300 IR five-grass-pollen SLIT tablet’s sustained efficacy and efficacy after discontinuation of treatment was investigated by Didier et al. in a 5-year DBPC RCT in adults with AR at centres in the European Union and Canada [39,52,53]. In fact, the European Medicines Agency currently requires AIT preparations to have (i) efficacy in the first season after the start of treatment, (ii) a proven, sustained clinical effect (defined as the maintenance of significant and clinically relevant efficacy during two to three treatment years) and (iii) long-term efficacy (defined as sustained significant and clinically relevant efficacy in post-treatment years, i.e. a disease-modifying effect) [65].

Hence, Didier et al. investigated three seasons of pre-seasonal and co-seasonal treatment with a 300 IR five-grass-pollen SLIT tablet in adult patients (aged 18 to 50) with a history of seasonal AR to grass pollen for more than two pollen seasons and continued to monitor the patients for a further two treatment-free seasons [39,52,53,66]. A total of 633 patients were randomized into one of three groups: (i) 300 IR grass pollen SLIT tablet, with active treatment starting 4 months (the 4 M group) before the expected start of the pollen season followed by co-seasonal treatment; (ii) 300 IR grass pollen SLIT tablet, with first a placebo taken for 2 months (starting 4 months before the expected start of the pollen season, to maintain the blinding), followed by 2 months of active treatment (the 2 M group) and then co-seasonal active treatment, and lastly (iii) placebo treatment starting 4 months before the expected start of the pollen season, followed by co-seasonal treatment. These discontinuous treatments were administered for three pollen seasons (years 1 to 3). After the end of the third treatment season, patients were monitored over the following two treatment-free pollen season (years 4 and 5). Of the 633 randomized patients, 457 completed year 3 and 435 contributed to the (post-treatment) efficacy analyses in year 4. The study’s primary efficacy end-point was a symptom score over the third pollen period. Secondary endpoints included the six individual rhinoconjunctivitis symptom scores (sneezing, rhinorrhoea, nasal pruritus, nasal congestion, ocular pruritus and watery eyes), the average medication score and the RQLQ score. In year 4, the symptom scores in the two active treatment groups were similarly and significantly lower than in the placebo group, and significant efficacy was determined in year 5 [66]. In a subsequent *post hoc* analysis, the mean LS daily combined score was found to be 28.1% lower in the 300 IR tablet group than the placebo group p = 0.0478), thus demonstrating long-term efficacy and a disease-modifying effect of the 300 IR five-grass-pollen SLIT tablet. The difference in LS means between the 4 M group and the 2 M group was not statistically significant. The 300 IR five-grass-pollen SLIT tablet’s safety profile over the entire study period was consistent with previous reports, with generally mild, transient, local AEs [39,52,53,67]. Over the first three treatment seasons, the incidence and severity of TEAEs decreased from one year to the next in all three treatment groups. In the first treatment season, the most frequently reported TEAEs were oral pruritus (30% and 11.4% in the active and placebo groups, respectively), throat irritation (15% and 3.7%, respectively), and mouth oedema (6% and 1.4%, respectively). During the first treatment year (but not the second and the third treatment year), the number of discontinuations due to TEAEs was higher in patients treated with the 300 IR formulation (7.2% in the two active groups and 1.4% in the placebo group). During the fourth year of study, the incidence of treatment-emergent AEs was similar in the 300 IR (4 M), 300 IR (2 M) and placebo groups, with values of 31.7%, 34.8% and 35.9%, respectively. Importantly, exacerbation of asthma was not an issue, and the one case in the 300 IR (4 M) and the two cases in the placebo group were not judged to be related to the study treatment [52,53].

Following on from the above-mentioned DBPC RCTs, a number of post-marketing studies in Europe have confirmed the 300 IR five-grass-pollen SLIT tablet’s safety and/or efficacy when a pre- and co-seasonal regimen is used in actual clinical practice. In Germany, the OPTIMAL multicentre, prospective, open-label, non-interventional study assessed the tolerability and effectiveness of two consecutive years of a pre- and co-seasonal treatment regimen with the 300 IR five-grass-pollen SLIT tablet in a total of 1,482 adult and paediatric patients (including 248 children aged from 4 to 11 and 201 adolescents aged from 12 to 17) [68]. Compared with the baseline season, the mean rhinoconjunctivitis scores were significantly lower in the two treatment seasons (by 51% and 64%, respectively; p < 0.001). Importantly, the asthma symptom score for the 522 patients with comorbid mild asthma was also significantly lower in the two treatment seasons (by 60% and 70%, respectively for year 1 and 2, respectively; p < 0.001). When considering the children alone, the mean rhinoconjunctivitis score fell from 4.1 in the baseline season to 1.93 after the first season and 1.39 after the second. For the adolescent subgroup, the corresponding values were 4.05, 1.97 and 1.39. The mean rhinoconjunctivitis score fell in both mono-allergic and polyallergic subjects, confirming the DBPC RCT results. For example, the respective baseline, year 1 and year 2 rhinoconjunctivitis score were 4.05, 1.95 and 1.29 in mono-allergic children and 4.09, 1.94 and 1.46 in polyallergic children [68].

A similar multicentre, open-label, observational, cross-sectional, single-season study of pre- and co-seasonal treatment with a 300 IR five-grass-pollen SLIT tablet has been carried out in Spain [69]. A total of 226 adult patients (mean age: 33.9 ± 11.5) were included in the study. Fifty-five percent had mild asthma and 92% of patients had persistent moderate-severe AR (according to the Allergic Rhinitis and its Impact on Asthma criteria [1,2]). Following pre- and co-seasonal treatment with the 300 IR five-grass-pollen SLIT tablet, the percentage of patients with moderate-severe AR was 30%. Symptomatic medication use also fell; the percentages of patients using oral H1-antihistamines and ICSs decreased from 92% and 77%, respectively, at baseline to 62% and 28%, respectively, at the end of the treatment period [69].

## Post-marketing safety profile of the five-grass pollen SLIT tablet

In Germany, post-marketing authorization safety studies of pre- and co-seasonal treatment with a 300 IR five-grass-pollen SLIT tablet have been carried out in children and adults [39,59,60,70]. In one study, 354 study centres across Germany enrolled 808 adults and 91 children and adolescents with grass pollen-induced AR and/or allergic conjunctivitis [39]. After a mean treatment period of 191 days, 320 patients (35.6%) reported at least one AE. The most common AEs were coded as oral paresthesia (10.9% of events), oral pruritus (6.9%), throat irritation (7.7%) and mouth oedema (5.3%). Eighty-five patients (9.5%) withdrew from treatment as a result of AEs.

In a second study, 207 centres enrolled 829 children and adolescents aged between 5 and 17 years [70]. After a mean treatment period of 190 days, 218 patients (27.4%) reported at least one AE (mainly throat irritation (n = 114), oral paresthesia (n = 49), oral pruritus (n = 39) and mouth oedema (n = 37). The most serious AE (Quincke’s oedema of the lips) occurred in a patient having developed angioedema during a course of SCIT a year earlier. Tolerability was rated as good or very good by 84.7% of the patients, 87.0% of the parents and 89.7% of the clinicians. Neither anaphylaxis nor adrenalin use was recorded in these two studies [39,70]. Lastly, in a 2-year study based on grass pollen AIT renewal rates, the persistence of treatment was significantly better in patients receiving SLIT than in those receiving SCIT (p < 0.02) [55]. Hence, pre & co-seasonal (discontinuous) treatment with a 300 IR five-grass-pollen SLIT tablet shows good effectiveness and a good safety profile in real-life clinical practice.

## Comparisons of the efficacy and safety of pre-/co-seasonal and perennial SLIT regimens

Few studies have performed head-to-head comparisons of a pre- and co-seasonal regimen and a perennial regimen. Obviously, single- or double-blind trials are difficult to perform if the durations of the compared treatment regimens are clearly different (unless patients in the pre-and-co-seasonal treated cohorts receive placebo after the end of the season). A study by Sieber et al. encompassed an independent patient data meta-analysis of three open, prospective, observational trials of standardized Pooideae family or Betulaceae family pollen SLIT preparations in a total of 1052 patients with pollen-induced AR [61]. The objective was to indirectly compare perennial treatments with co-seasonal treatments and compare ultra-rush titration with standard titration. The three studies respectively featured a perennial regimen with classical titration, a co-seasonal regimen with ultra-rush titration and either a perennial or a co-seasonal regimen. The most frequently used allergen preparations contained Betulaceae pollen extracts (49.9%) and Pooideae pollen extracts (41.6%). Overall, the various treatment regimens were all associated with improvements in symptom scores and medication scores (when comparing the study period to a baseline period) and there were no significant differences between the regimens. Sieber et al. suggested that the equivalent effectiveness of co-seasonal and perennial SLIT treatment might have an impact on the yearly cost of treatment, although compliance was not assessed [61].

Quercia performed a head-to-head study of a pre- and co-seasonal regimen with a solely pre-seasonal regimen [71]. The SLIT product was a tablet formulation of grass pollen monomeric carbamylated allergoids. Three groups of patients were studied. Group 1 took a 1,000 allergenic unit (AU) tablet once a week continuously from November 2005 to July 2007 (i.e. through two grass pollen seasons). Group 2 took a 1,000 AU tablet five times a week during each 10-week pre-seasonal period solely. Both SLIT groups showed a similar and significant improvement on a visual analogue scale of symptom severity after the first and second pollen seasons, relative to the baseline symptoms in Groups 1 and 2 and also the symptoms of a third group of patients (Group 3) taking symptomatic medications only. The study had several limitations. Firstly, it used an open design, although it is hard to conceive a fully double-blind, controlled, randomized trial in which some patients (but not others) are required to stop taking a treatment for a certain period of time. Secondly, the study subgroups were small (n = 10, 11 and 11 in Groups 1 to 3, respectively). Thirdly, Groups 1 and 2 did not receive the same dose of allergen (on a per-week basis) in the presumably crucial pre-seasonal period.

There are no head-to-head comparative studies of the safety of a pre- and co-seasonal regimen vs. a year-round regimen. However, in the pivotal European and North America clinical trials of the grass pollen SLIT tablets, the single-grass formulation and the five-grass formulation did not appear to have radically different safety profiles. Nelson et al. [37] commented that although the majority of adult and paediatric patients (60-80%) taken the single-grass tablet experienced adverse events, the latter were generally local, mild-to-moderate and transient. The most common local AEs were oral pruritus (observed in 32-54% of patients on active treatment in the various studies) and throat irritation (in 9-37%). Systemic adverse events were rare [37]. When summarizing the safety data for the five-grass tablet, Didier et al. [39] used very similar language. Most AEs during treatment were mild or moderate in severity, with oral pruritus and throat irritation again being among the most common (reported in >5% of patients) [39]. No deaths or anaphylactic reactions were observed in these trials.

In view of the published evidence, a pre-seasonal and co-seasonal regimen appears to be sufficient for demonstrating the efficacy and safety of SLIT formulations of a 300 IR birch pollen extract and a 300 IR five-grass extract. On this basis, one can question whether year-round SLIT regimens for pollen-induced AR have added clinical or economic value. Indeed, a 75,000 SQ-T single-grass SLIT tablet registered with a year-round regimen has been successfully applied in off-label pre- and co-seasonal use [72]. In a multicentre, prospective, open-label, observational study in France, 130 physicians treated a total of 628 patients for an average of 5.5 months per year (with 4 months of pre-seasonal treatment and then co-seasonal treatment) for three successive seasons. Efficacy data were not reported, since safety was the primary study criterion. Treatment-related AEs were reported for respectively 46.2%, 14.4% and 1.8% of the patients during the first, second and third years of the study. The compliance rate was 71.8% in the first year, 86.8% in the second year and 90.3% in the third year – again demonstrating the good compliance observed with pre- and co-seasonal regimens [72]. Data from 934 patients treated with the 75,000 SQ-T single-grass SLIT tablet (registered for use with a year-round regimen) have also been used to show that at least 2 months of pre-seasonal treatment are required for clinical efficacy [54].

## Relationships between allergen dose and the administration regimen

There are probable relationships between the maintenance dose of allergen, clinical efficacy and the administration regimen. It is now beyond doubt that AIT involves a dose-response relationship. Of fifteen dose-ranging studies reviewed by the European Academy of Allergy and Clinical Immunology’s Task Force Report on Dose-Response Relationship in Allergen-Specific Immunotherapy [73], twelve reported a dose-response relationship for clinical efficacy. In this respect, one can consider the above-mentioned 300 IR five-grass-pollen SLIT tablet and 75,000 SQ-T single-grass-pollen SLIT tablet. According to the standardized unit for allergen extracts required by the US Food and Drug Administration (the bioequivalent allergy unit, BAU, based on the reaction to an intradermal test in highly allergic patients) [74], the 300 IR five-grass-pollen SLIT tablet contains 9,000 BAU [75] and the 75,000 SQ-T single-grass-pollen SLIT tablet contains 2,800 BAU [76]. According to the respective manufacturers’ in-house assays, the major allergen (Phl p 5) content is 25 μg for the 300 IR five-grass-pollen SLIT tablet and 15 μg for the 75,000 SQ-T single-grass-pollen SLIT tablet [64,77]. Although these tablets differ in terms of BAUs and major allergen content (for optimal comparisons, these parameters should be determined by the same laboratory in the same patients and using the same reagents [74,78]), the two formulations appear to provide similar efficacy in the first year of Phase III trials (as assessed by the effect size for the rhinoconjunctivitis score, relative to placebo) [36-39]. When considering pre- and co-seasonal regimens with grass-pollen tablets, only the 300 IR five-grass formulation has proven sustained and post-treatment efficacy in addition to single-season efficacy [39]. Furthermore, a lower-dose (100 IR) five-grass-pollen SLIT tablet was no more effective than placebo in the pivotal, dose-ranging RCT [64].

## The health economics of pre- and co-seasonal regimens

Pre- and co-seasonal treatment regimens have economic and compliance benefits relative to perennial regimens. Firstly, a shorter duration of treatment of discontinuous protocols implies that patients will receive less medication, thus leading to a lower acquisition cost of medication. Two studies evaluated the cost-effectiveness of pre- and co-seasonal SLIT in grass pollen-induced AR [79,80].

Westerhout et al. undertook a cost-effectiveness analysis of 3 years of treatment with a pre- and co-seasonal 300 IR 5-grass pollen SLIT tablet (in addition to symptomatic drug treatment (SDT)) over a time horizon of 9 years, compared with the following: (i) perennial SLIT with a single grass tablet (in addition to SDT), (ii) perennial SCIT regimen (in addition to SDT), and (iii) SDT alone (i.e. standard of care, SoC) for the treatment of grass pollen-induced AR from the German health care perspective [79]. Treatment efficacy was estimated based on an indirect comparison of randomized controlled trials (RCTs) with placebo (i.e. SoC) as a common comparator. Moreover, a meta-analysis of RCTs of the three treatment regimens was conducted to determine the effectiveness of each treatment relative to placebo (i.e. SoC) for reducing AR symptom scores and improving the number of symptom-free days. The results indicated that relative to SDT alone, the incremental cost per quality-adjusted life-year (QALY) gained with 300 IR 5-grass pollen SLIT was €14,728, with incremental costs of €1,356 and incremental QALYs of 0.092. The resulting incremental cost-effectiveness ratio falls below commonly accepted thresholds in Europe. Meanwhile, relative to perennial single grass SLIT tablet or perennial SCIT, estimated incremental cost savings with five-grass-pollen SLIT tablet were respectively €1,142 and €54, and the corresponding incremental QALYs were respectively 0.015 and 0.027. Hence, the results indicate that the pre- and co-seasonal 5-grass pollen SLIT tablet is a cost-effective treatment option (relative to perennial single grass SLIT tablet, perennial SCIT and SDT alone) for the treatment of grass pollen-induced allergic rhinitis in Germany. However, extensive sensitivity analyses indicated that the model’s outcomes may have been affected by uncertainty surrounding treatment efficacy estimates [79].

In a recent economic study [80], a cost-minimization analysis was conducted on the basis of a systematic review of 20 DBPC trials in grass pollen-induced seasonal AR. The following four treatment regimens from the Canadian health care perspective: (i) pre- and co-seasonal treatment with a 300 IR five-grass-pollen SLIT tablet, together with SDT if required, and (ii) perennial SLIT with a single-grass tablet, (iii) a perennial SCIT regimen, and (iv) a pre- and co-seasonal SCIT regimen. The results indicated that the pre- and co-seasonal 300 IR five-grass-pollen SLIT regimen has at least non-inferior efficacy, similar safety and a lower annual cost, when compared with perennial or seasonal SCIT, and perennial SLIT with a single grass tablet [80]. During the first year of treatment, pre-and co-seasonal administration of the 300 IR five-grass pollen SLIT tablet plus SDT was associated with significant cost savings relative to perennial SCIT (Can $2,471), seasonal SCIT (Can $948), and perennial SLIT with a single-grass tablet (Can $1,168) [80].

## Conclusion

In pollen-induced AR, discontinuous SLIT regimens appear to be at least as effective and safe as perennial SLIT regimens in terms of sustained efficacy over consecutive pollen seasons, and even after the discontinuation of treatment. Head-to-head studies are required to establish whether discontinuous SLIT regimens are associated with lower costs and/or better compliance or safety than perennial SLIT regimens. When considering the pre- and co-seasonal discontinuous regimen in particular, a 300 IR five-grass-pollen formulation is the only SLIT tablet with a clinical development programme having provided evidence of short-term, sustained and post-treatment efficacy.

## Abbreviations

AE: Adverse event
AIT: Allergen immunotherapy
AR: Allergic rhinitis
BAU: Bioequivalent allergy unit
DBPC: Double-blind, placebo-controlled
ICS: Intranasal corticosteroid
IgE: Immunoglobulin E
IR: Index of reactivity
LS: Least-squares
M: Month
QALY: Quality-adjusted life year
RCT: Randomized clinical trial
RQLQ: Rhinoconjunctivitis quality of Life Questionnaire
SCIT: Subcutaneous immunotherapy
SDT: Symptomatic drug treatment
SLIT: Sublingual immunotherapy
SoC: Standard of care
SQ-T: Standardized quality tablet
TEAE: Treatment-emergent adverse event
